# Genetic diversity, haplotypes and allele groups of Duffy binding protein (PkDBPαII) of *Plasmodium knowlesi* clinical isolates from Peninsular Malaysia

**DOI:** 10.1186/1756-3305-7-161

**Published:** 2014-04-03

**Authors:** Mun-Yik Fong, Yee-Ling Lau, Phooi-Yee Chang, Claudia Nisha Anthony

**Affiliations:** 1 Department of Parasitology, Faculty of Medicine, University of Malaya, 50603 Kuala Lumpur, Malaysia; 2 Tropical Infectious Diseases Research and Education Centre (TIDREC), Faculty of Medicine, University of Malaya, 50603 Kuala Lumpur, Malaysia

**Keywords:** *Plasmodium knowlesi*, Duffy binding protein, Diversity, Selection, Haplotypes, Allele groups

## Abstract

**Background:**

The monkey malaria parasite *Plasmodium knowlesi* is now recognized as the fifth species of *Plasmodium* that can cause human malaria. Like the region II of the Duffy binding protein of *P. vivax* (PvDBPII), the region II of the *P. knowlesi* Duffy binding protein (PkDBPαII) plays an essential role in the parasite’s invasion into the host’s erythrocyte. Numerous polymorphism studies have been carried out on PvDBPII, but none has been reported on PkDBPαII. In this study, the genetic diversity, haplotyes and allele groups of PkDBPαII of *P. knowlesi* clinical isolates from Peninsular Malaysia were investigated.

**Methods:**

Blood samples from 20 knowlesi malaria patients and 2 wild monkeys (*Macaca fascicularis*) were used. These samples were collected between 2010 and 2012. The PkDBPαII region of the isolates was amplified by PCR, cloned into *Escherichia coli*, and sequenced. The genetic diversity, natural selection and haplotypes of PkDBPαII were analysed using MEGA5 and DnaSP ver. 5.10.00 programmes.

**Results:**

Fifty-three PkDBPαII sequences from human infections and 6 from monkeys were obtained. Comparison at the nucleotide level against *P. knowlesi* strain H as reference sequence showed 52 synonymous and 76 nonsynonymous mutations. Analysis on the rate of these mutations indicated that PkDBPαII was under purifying (negative) selection. At the amino acid level, 36 different PkDBPαII haplotypes were identified. Twelve of the 20 human and 1 monkey blood samples had mixed haplotype infections. These haplotypes were clustered into 2 distinct allele groups. The majority of the haplotypes clustered into the large dominant group.

**Conclusions:**

Our present study is the first to report the genetic diversity and natural selection of PkDBPαII. Hence, the haplotypes described in this report can be considered as novel. Although a high level of genetic diversity was observed, the PkDBPαII appeared to be under purifying selection. The distribution of the haplotypes was skewed, with one dominant major and one minor group. Future study should investigate PkDBPαII of *P. knowlesi* from Borneo, which hitherto has recorded the highest number of human knowlesi malaria.

## Background

Malaria is caused by blood protozoa of the genus *Plasmodium.* Historically, four species of *Plasmodium* are known to cause human malaria: *Plasmodium falciparum*, *P. vivax*, *P. malariae*, and *P. ovale*. However, about a decade ago, *P. knowlesi*, a malaria parasite of macaque monkeys, was reported to cause a large number of human infections in Sarawak, Borneo Island [1]. Since then, human knowlesi malaria has been reported in other parts of Borneo Island, Peninsular Malaysia, and in many other countries in Southeast Asia [2]. Imported knowlesi malaria cases have been reported in European countries and Japan due to eco-tourism programs to the forested areas of Southeast Asia. Now, *P. knowlesi* is recognised as the fifth species of *Plasmodium* that can cause human malaria.

Invasion of a malaria parasite into its host erythrocyte depends on the interaction between the parasite’s protein and the corresponding receptor on the surface of the erythrocyte. *Plasmodium vivax* and *P. knowlesi* use the Duffy blood group antigen as a receptor to invade erythrocytes [3]. The Duffy binding proteins of *P. vivax* (PvDBP) and *P. knowlesi* (PkDBP) are located on their merozoites. PvDBP and PkDBP are members of the erythrocyte-binding protein family which also includes the *P. falciparum* EBA-175 [4]. PvDBP and PkDBP are large proteins and can be divided into seven regions (I-VII). Region II contains the critical motifs for binding to the erythrocyte Duffy antigen.

PkDBP is encoded by an α-gene and therefore is more specifically known as PkDBPα. This is to distinguish it from two other highly homologous proteins in *P. knowlesi* – β and γ. Region II of the β and γ proteins have different binding specificities compared to PkDBPα. Region II of the β and γ proteins binds to rhesus erythrocytes but does not bind to Duffy antigen of human erythrocytes [5].

PvDBP has been suggested to be an important vaccine candidate antigen against vivax malaria because it elicits strong immune responses in humans. Design of vaccine against vivax malaria must take into consideration the nature and genetic polymorphism of PvDBP. Region II of PvDBP (denoted as PvDBPII) in *P. vivax* isolates from different geographical regions such as Colombia, South Korea, Papua New Guinea, Thailand, Iran and Myanmar has been shown to be highly polymorphic, and numerous PvDBPII haplotypes and allele groups have been identified [6-11].

It has been observed that antibodies raised against PkDBPαII could inhibit *P. knowlesi* invasion of human and rhesus erythrocytes *in vitro*[12]. Therefore, like PvDBPII for vivax malaria, PkDBPαII may also be a candidate vaccine antigen against knowlesi malaria. Whilst many polymorphism studies on PvDBPII have been carried out, hitherto, none has been reported for PkDBPαII. In this report, we present our findings on genetic diversity, haplotyes and allele groups of PkDBPαII in *P. knowlesi* clinical isolates from Peninsular Malaysia.

## Methods

### Blood samples

Human blood samples used in this study were collected from 20 patients who were infected with *Plasmodium knowlesi* at the University of Malaya Medical Centre and several private clinics in Peninsular Malaysia. Two blood samples from *P. knowlesi*-infected wild monkeys (*Macaca fascicularis*) were provided by the Wildlife Department of Federal Territory, Peninsular Malaysia. These blood samples were collected between 2010 and 2012 from various states in Peninsular Malaysia (Table 1). Confirmation of *P. knowlesi* infection in all the samples was carried out by microscopic examination of Giemsa-stained thin and thick blood smears and polymerase chain reaction. Ethical approvals for the use of human and monkey blood samples in this study were granted by the University of Malaya Medical Centre Ethic Committee and (MEC No. 817.18) and the University of Malaya Animal Care and Use Committee (PAR/19/02/2013/AA[R]).

**Table 1:** Date and origin of human and monkey blood samples used in this study

**Blood sample**	**Date (Month, year)**	**Origin (State in Peninsular Malaysia)**
ANU	Feb 2012	Kelantan
AZL	Jun 2011	Perak
CHO	Oct 2010	Pahang
GAN	Oct 2011	Selangor
HAI	Feb 2012	Kelantan
HAN	Feb 2012	Kelantan
HEN	Feb 2012	Kelantan
IZA	Aug 2010	Selangor
JUN	Feb 2012	Kelantan
MAD	Aug 2010	Negeri Sembilan
MAH	Aug 2011	Selangor
MEL	Feb 2012	Kelantan
NGO	Oct 2010	Perak
OTH	Feb 2012	Kelantan
RAU	Feb 2012	Kelantan
SUP	Feb 2012	Kelantan
SYA	Feb 2012	Kelantan
UM0001	Jan 2012	Selangor
UM0002	Feb 2012	Kelantan
UM0004	Apr 2012	Selangor
Monkey566	Oct 2010	Negeri Sembilan
Monkey569	Oct 2010	Pahang

### Extraction of DNA

Total DNA of the *P. knowlesi* was extracted from each blood sample using the QIAGEN Blood DNA Extraction kit (QIAGEN, Hilden, Germany). In each extraction, 100 μl of blood was used. The extracted DNA was suspended in water to a final volume of 50 μl.

### PCR, cloning and sequencing of the PkDBPαII

The PkDBPαII was amplified by nested PCR using oligonucleotide primers Pkα-DBP-F1: 5′-CGCATTTTGAAGGAATCCAC-3′ and Pkα-DBP-R1: 5′-TGCTAGACTTACCTTCACCT-3′ for nest 1. The primers for the nest 2 reaction were Pkα-DBP-F: 5′-TCCTCAAAAGGCGGTGACCATCC-3′ and Pkα-DBP-R: 5′-ACTGGCTGCCTTAGATTCAACACCA-3′. Cycling conditions for nest 1 were as follows: 95°C for 4 mins, 30 cycles at 95°C for 30 secs, 48°C for 30 secs, and 72°C for 90 secs, followed by a 10 min extension at 72°C. The amplification for nest 2 was performed using the following cycling profile: 95°C for 4 mins, 30 cycles at 95°C for 30 secs, 56°C for 30 secs, and 72°C for 90 secs, followed by a 10 min extension at 72°C. The PCR product with an expected size of 1053 bp was analyzed on a 1% agarose gel.

### Purification of PCR products and DNA cloning

PCR products were purified by QIAquick PCR purification Kit (QIAGEN, Hilden, Germany) following the manufacturer’s instructions. The purified PCR products were then ligated into cloning vector pGEM-T^®^ (Promega Corp., USA) and transformed into *Escherichia coli* TOP10F’. Plasmids of recombinant clones harbouring the PkDBPαII fragment were sent to a commercial laboratory for DNA sequencing. To detect the possibility of multiple haplotypes infecting a patient or monkey, plasmids from 4–6 recombinant clones from each transformation mixture were sequenced.

### Analysis of PkDBPαII sequences

Alignment of 60 sequences of PkDBPαII [53 of human origin, 6 of monkey origin, 1 reference strain (strain H, GenBank Accession No. M90466)] were performed using CLUSTAL-Omega programme which was available on-line (http://www.ebi.ac.uk/Tools/msa/clustalo). Both nucleotide and the deduced amino acid sequences were aligned and analysed. Phylogenetic tree was constructed using the Neighbour Joining method described in MEGA5 [13]. In constructing the phylogenetic tree, bootstrap replicates of 1000 were used to test the robustness of the tree.

### PkDBPαII sequence polymorphism analysis

DnaSP ver. 5.10.00 [14] was used to perform polymorphism analysis on the 60 PkDBPαII sequences. Information such as the number of segregating sites (S), haplotype diversity (Hd), nucleotide diversity (π), and average number of pairwise nucleotide differences within the population (K) were generated. The π was also calculated on a sliding window of 100 bases, with a step size of 25 bp to estimate the stepwise diversity across PkDBPαII. The rates of synonymous (*K*s) and non-synonymous (*K*n) substitutions were estimated and compared by the Z-test (P < 0.05) in MEGA5 using the Nei and Gojobori’s method [15] with the Jukes and Cantor correction. In the case of purifying (negative) selection, mutations are usually not advantageous so that *K*n will be less than *K*s (*K*n/*K*s < 1). However, in positive selection, non-synonymous mutations can be advantageous and *K*n will exceed *K*s (*K*n/*K*s > 1). For testing the neutral theory of evolution, Tajima’s D [16] and Fu and Li’s D and F [17] tests was carried out using DnaSP 5.10.00. In the Fu and Li’s tests, *P. vivax* PvDBPII (GenBank Accession No. M90466) was used as outgroup.

## Results

Nested PCR amplification on the human and monkey blood samples isolates produced DNA fragments of 1053 base pairs. The sequence of each fragment was trimmed to 921 bp, based on the PkDBPαII region identified by Singh *et al*. [18]. This trimmed sequence encoded an amino acid sequence of 307 in length. A final total of 59 sequences (GenBank Accession No. KC597079 – KC597137) were obtained.

**Figure 1:**
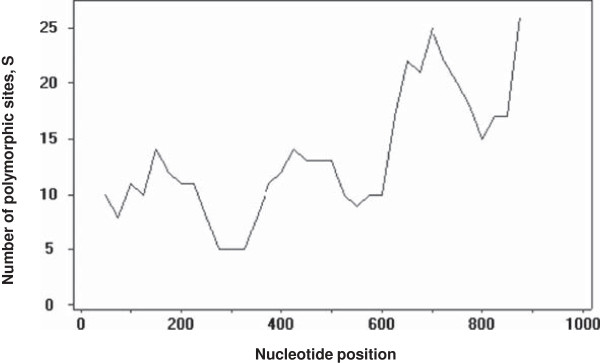
**Nucleotide polymorphism in the PkDBPαII of *P. knowlesi. ***Sliding window plot of number of polymorphic sites (S) along the PkDBPαII, generated by using DnaSP ver. 5.10.00 with a window length of 100 bp and step size of 25 bp.

DNA sequence analyses were conducted to determine nucleotide diversity and genetic differentiation. The average number of pairwise nucleotide differences (K) for the PkDBPαII was 11.736. The overall haplotype diversity (Hd) and nucleotide diversity (π) for the 60 PkDBPαII sequences were 0.986 ± 0.008 and 0.013 ± 0.002, respectively. Detailed analysis of π, with a sliding window plot (window length 100 bp, step size 25 bp), revealed diversity ranged from 0.003 to 0.034. The highest peak of nucleotide diversity was within nucleotide positions 600–750, whereas the most conserved region was within nucleotide positions 260–360 (Figure 1).

Analysis and comparison at the nucleotide level against *P. knowlesi* strain H as reference sequence showed mutations at 128 positions among the Peninsular Malaysia isolates. Fifty-two of these mutations were synonymous and 76 were nonsynonymous. To determine whether natural selection contributed to the diversity in the PkDBPαII, the rate of nonsynonymous (*K*n) to synonymous mutations (*K*s) was estimated. *K*n (0.00952) was found lower than *K*s (0.00278) and the *K*n/*K*s ratio was 0.347, suggesting that purifying (negative) selection may be occurring in the PkDBPαII. Similarly, the Z test (*K*s > *K*n; P < 0.05) also indicated purifying selection on PkDBPαII. In the tests of departure of neutrality of selection, the Tajima’s D statistics was -2.085 (P < 0.05), indicating expansion in population size and/or purifying selection. This is further supported by the Fu and Li’s D and F tests statistics (-3.772 and -3.756, respectively; P < 0.05).

**Figure 2:**
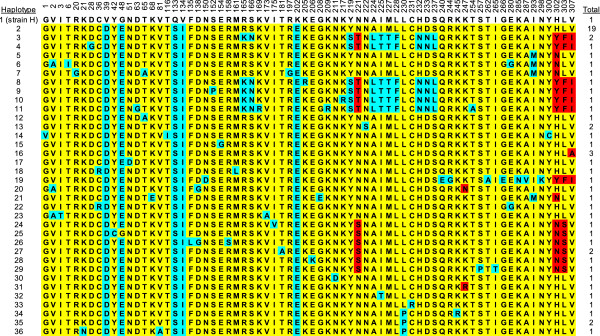
**Amino acid sequence polymorphism in PkDBPαII. **Polymorphic amino acid residues are listed for each haplotype. Amino acid residues identical to those of the reference sequence, strain H (haplotype 1), are marked in yellow. Monomorphic and dimorphic amino acid changes are marked in blue and red, respectively. Total number of sequences for each haplotype is listed in the right panel.

**Figure 3:**
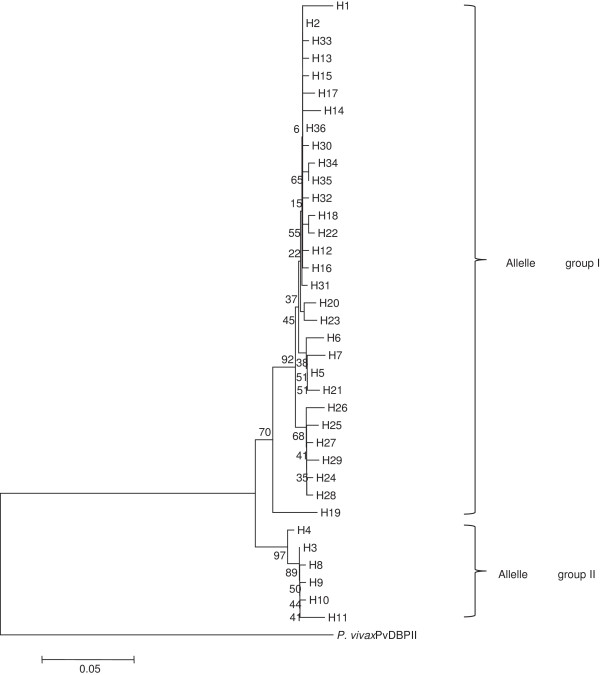
**Phylogenetic tree of PkDBPαII haplotypes. **Neighbour-Joining method was used to construct the tree, which contains 36 haplotypes. *Plasmodium vivax *PvDBPII was used as outgroup. Numbers at nodes indicate percentage support of 1000 bootstrap replicates.

Comparison at the amino acid level revealed high polymorphism across the entire PkDBPαII of the isolates (Figure 2). Among the 70 polymorphic sites, 66 showed monomorphic mutation (changed into one amino acid-type) and 4 showed dimorphic mutation [changed into two amino acid types: 221 (Q, T), 302 (N, Y), 303 (F, S), 307 (A, I)]. The amino acid sequences could be categorised into 36 different haplotypes (H1-H36) with haplotype 2 having the highest frequency (19/60). Twelve of the 20 human and 1 monkey blood samples had mixed haplotype infections (Table 2). Phylogenetic tree analysis revealed that the haplotypes could be clustered into 2 main allele groups (Figure 3).

**Table 2:** PkDBPαII haplotypes detected in the human and monkey blood samples

**Blood sample**	**Haplotype**
ANU	H2
AZL	H9, H10, H11
CHO	H3, H8
GAN	H5, H6, H7
HAI	H18, H19
HAN	H2
HEN	H2
IZA	H2, H3, H4
JUN	H15
MAD	H24, H25, H26, H27, H28, H29
MAH	H2, H33
MEL	H12, H13, H14
NGO	H2
OTH	H2, H16
RAU	H2, H30, H31
SUP	H17
SYA	H2, H20
UM0001	H21
UM0002	H2, H22, H23
UM0004	H32
Monkey566	H2
Monkey569	H34, H35, H36

## Discussion

The DBP of *P. vivax* and *P. knowlesi* play an essential role in erythrocyte invasion of the parasites by mediating irreversible binding with its corresponding receptor, the Duffy protein receptor for chemokines (DARC) on the surface of erythrocytes [19]. The DBP elicits strong immune responses in humans and therefore has been suggested to be an important vaccine candidate antigen [12,20]. The critical erythrocyte-binding motif of DBP has been identified to be located in region II of the protein. The *P. vivax* PvDBP has been observed to have a high degree of genetic polymorphism, and most of the polymorphism is located in region II. Although the cysteine and some hydrophobic amino acid residues in PvDBPII are conserved in *P. vivax* populations from different geographic regions, but the remaining amino acid residues are highly polymorphic [6-11,21-24]. It has further been revealed that PvDBPII undergoes positive natural pressure which results in allelic variation as a mechanism for immune evasion. On the other hand, despite the emerging importance of *P. knowlesi* as a human pathogen, no study has thus far been carried out on PkDBPαII. Our present study is the first to report the diversity and natural selection of PkDBPαII.

The PkDBPαII analysed in this study is based on the region defined by Singh *et al*. [18] using structural biology analysis. In their analysis, twelve C residues (positions 16, 29, 36, 45, 99, 176, 214, 226, 231, 235, 304, 306), which form six disulphide bridges, have been suggested to be involved in the folding of PkDBPαII for interaction with DARC. Multiple alignment of the 60 PkDBPαII amino acid sequences (Additional file 1) in our study revealed that these 12 residues were highly conserved. Apart from these conserved C residues, the Y94, N95, K96, R103, L168 and I175 residues are required for recognition of DARC on human erythrocytes [18]. Again, our multiple sequence alignment showed high conservation of these residues in the PkDBPαII. In fact, Y94, N95, K96 and R103 fall within the most conserved region in the PkDBPαII (nucleotide positions 260–360 in the gene, Figure 1).

The PkDBPαII (K = 11.736; Hd = 0.986; π = 0.01274) in Peninsular Malaysia seemed to be more diverse than *P. vivax* PvDBPII in some neighbour countries, such as Myanmar (K = 7.851; Hd = 0.875; π = 0.00790), Korea (K = 2.878; Hd = 0.775; π = 0.00299) and Sri Lanka (Hd = 0.922; π = 0.00982) [7,11,25]. Generally, high levels of genetic diversity in malaria parasite surface antigens are attributed to positive natural selection, for example, by the host immune system [26]. Positive selection has been reported in PvDBPII of isolates in Myanmar, Korea, Sri Lanka and Thailand [7,9,11,25]. On the contrary, the PkDBPαII in Peninsular Malaysia was found to be under purifying (negative) selection. The reason for this finding is unclear, but may be associated with the host of the parasite. For example, it has been reported that the rhoptry-associated protein 1 (RAP-1) antigen of non-human primate malarial parasites (*P. knowlesi, P. cynomolgi, P. inui* and *P. fieldi*) showed evidence for negative selection, which was not found in two human malarial parasites (*P. falciparum, P. vivax*) [27]. Another possible reason for the purifying selection on PkDBPαII is population expansion of *P. knowlesi*, as evident by the Tajima’s D statistics. In Peninsular Malaysia, deforestation in many areas has increased monkey and forest dwelling *Anopheles* vector contact to human. The expansion of monkey and vector populations and their ecological niches into human habitation may therefore result in the population expansion of *P. knowlesi*[28].

Phylogenetic studies on *P. vivax* isolates based on PvDBPII revealed the occurrence of multiple haplotypes in a geographical location, and these haplotypes could be categorized into several allele groups. For example, 25 haplotypes from Thailand were organized into 5 groups [9]. In Papua New Guinea, 27 haplotypes clustered into 3 dominant groups [9], whereas in Korea, 13 haplotypes were clustered into 3 groups [7]. Our study revealed the occurrence of 2 distinct allele groups of PkDBPαII in Peninsular Malaysia (Figure 3). Interestingly, allele group I was more dominant as it contained the majority (30/36) of the haplotypes. The minor allele group II had only 6 haplotypes. Two monkey blood samples were included in this study but all haplotypes from the monkeys were grouped in allele group I. The reference strain H, which was isolated in 1965 in Peninsular Malaysia from the first reported case of human *P. knowlesi* infection [29], was also grouped in allele group I. This shows that despite temporal separation of almost 50 years between strain H and the recent isolates of this study, no major genetic differences had occurred in the PkDBPαII.

## Conclusions

Our present study is the first to report the genetic diversity and natural selection of PkDBPαII. Hence, the haplotypes described in this report can be considered as novel. Although a high level of genetic diversity was observed, the PkDBPαII appeared to be under purifying (negative) selection. Two allele groups of haplotype were obtained. However, the distribution was skewed, in which majority of the haplotypes clustered in a large dominant group. Our study was carried out on Peninsular Malaysia isolates only. Therefore, future study should investigate PkDBPαII of *P. knowlesi* from Borneo, which hitherto has recorded the highest number of human knowlesi malaria.

## Competing interests

The authors declare that they have no competing interests.

## Authors’ contributions

MYF and YLL designed the study and supervised the study process. PYC performed all the experiments and analysed the sequence data. MYF and CNA performed sequence and phylogenetic analyses. MYF, YLL and CNA wrote the manuscript. All authors read and approved the final manuscript.

## Supplementary Material

Additional file 1 **Full amino acid sequence alignment of PkDBPαII.** Amino acid residues identical to those of the reference sequence (strain H), are indicated by dots. The twelve conserved cysteine (C) residues are marked in yellow. The conserved Y94, N95, K96, R103, L168 and I175 residues required for recognition of DARC on human erythrocytes are highlighted in green. Click here for file
