# Supplementary material for: Genetic diversity, haplotypes and allele groups of Duffy binding protein (PkDBPαII) of Plasmodium knowlesi clinical isolates from Peninsular Malaysia
Source: Parasit Vectors. 2014 Apr 3;7:161. doi: 10.1186/1756-3305-7-161 (PMC4022242; doi:10.1186/1756-3305-7-161)
Supplement: Additional file 1 — Full amino acid sequence alignment of PkDBPαII. Amino acid residues identical to those of the reference sequence (strain H), are indicated by dots. The twelve conserved cysteine (C) residues are marked in yellow. The conserved Y94, N95, K96, R103, L168 and I175 residues required for recognition of DARC on human erythrocytes are highlighted in green. [file 1756-3305-7-161-S1.doc]

**1111111111111111111111111111111111111111111111111111111111111111111111111111111111111111111111111111222222222222222222222222222222222222222222222222222222222222222222222222222222222222222222222222222233333333**

**1111111111222222222233333333334444444444555555555566666666667777777777888888888899999999990000000000111111111122222222223333333333444444444455555555556666666666777777777788888888889999999999000000000011111111112222222222333333333344444444445555555555666666666677777777778888888888999999999900000000**

**1234567890123456789012345678901234567890123456789012345678901234567890123456789012345678901234567890123456789012345678901234567890123456789012345678901234567890123456789012345678901234567890123456789012345678901234567890123456789012345678901234567890123456789012345678901234567890123456789012345678901234567**

**Strain H GVINQTFLQNNVMDKCNDKRKRGERDWDCPAEKDICISVRRYQLCMKGLTNLVNNTRTHSHNDITFLKLNLKRKLMYDAAVEGDLLLKKNNYQYNKEFCKDIRWGLGDFGDIIMGTNMEGIGYSQVVENNLRQVFGTDEKAKQDRKQWWNESKEHIWRAMMFSIRSRLKEKFVWICKKDVTLKVEPQIYRWIREWGRDYMSKLPKEQGKLNEKCASKLYYNNMAICMLPLCHDACKSYDQWITRKKKQWDVLSTKFSSVKKTQKIGTENIATAYDILKQELNGFKEATFENEINKRDNLYNHLCPCV**

**NGO-10 ......................................D........E....................................................................................SI...................................................................E.........................................................................................................**

**IZA-2 ......................................D........E....................................................................................SI..............................KN...................................E................S.T..LT.TF...NN...L................................................................YF.V.I**

**IZA-3.1 ......................................D........E....................................................................................SI...................................................................E.........................................................................................................**

**IZA-4.1 ...........................G..........D........E....................................................................................SI...................................................................E................S.T..LT.TF...NN...L................................................................YF.V.I**

**GAN-3 ......................................D........E....................................................................................SI...................................................................E..........................................................................................M........N.....**

**GAN-7 .A...I................................D........E....................................................................................SI...................................................................E.............................................................................G............M........N.....**

**GAN-9 ...................G..................D........E................A...................................................................SI..............................................................G....E..........................................................................................M........N.....**

**CHO-3 ......................................D........E....................................................................................SI..............................KN...................................E..R.............S.T..LT.TF...NN...L................................................................YF.V.I**

**CHO-11 ......................................D........E....................................................................................SI..............................KN...................................E................S.T..LT.TF...NN...L................................................................YF.V.I**

**AZL-2 ......................................D........E....................................................................................SI.................P............KN...................................E................S.T..LT.TF...NN...L................................................................YF.V.I**

**AZL-10 ......................................D........E....................................................................................SI..............................KN...................................E..............R.S.T..LT.TF...NN...L................................................................YF.V.I**

**AZL-14 ......................................D........E..............G.....................................................................SI..............................KN..R................................E......R.........S.T..LT.TF...NN...L................A...............................................YF.V.I**

**MEL-3 ......................................D........E................A...................................................................SI...................................................................E.........................................................................................................**

**MEL-4.1 ......................................D........E....................................................................................SI...................................................................E...................S.....................................................................................**

**MEL-5 ......................................D........E....................................................................................SI...................................................................E...................S.....................................................................................**

**MEL-11 V.....................................D........E...................................................................I................SI...................................................................E.................................................................................................C.......**

**JUN-11 ......................................D........E....................................................................................SI...................G...............................................E.........................................................................................................**

**HAN-7 ......................................D........E....................................................................................SI...................................................................E.........................................................................................................**

**HAN-10 ......................................D........E....................................................................................SI...................................................................E.........................................................................................................**

**HAN-14 ......................................D........E....................................................................................SI...................................................................E.........................................................................................................**

**OTH-1 ......................................D........E....................................................................................SI...................................................................E........................................................................................................A**

**OTH-3 ......................................D........E....................................................................................SI...................................................................E.........................................................................................................**

**OTH-7 ......................................D........E....................................................................................SI...................................................................E........................................................................................................A**

**OTH-8 ......................................D........E....................................................................................SI...................................................................E........................................................................................................A**

**ANU-6.1 ......................................D........E....................................................................................SI...................................................................E.........................................................................................................**

**ANU-7 ......................................D........E....................................................................................SI...................................................................E.........................................................................................................**

**SUP-4 ......S...............................D........E..D.................................................................................SI...................................................................E.........................................................................................................**

**HAI-1 ...................................R..D........E....................................................................................SI..........................L........................................E.........................................................................................................**

**HAI-5 ......................................D........E....................................................................................SI...............D...................................................E.....................................E...G.................A...E..................N.V..........K...YF.V.I**

**HEN-8 ......................................D........E....................................................................................SI...................................................................E.........................................................................................................**

**HEN-9 ......................................D........E....................................................................................SI...................................................................E.........................................................................................................**

**SYA-5 ......................................D........E....................................................................................SI...................................................................E.........................................................................................................**

**SYA-9 .A....................................D........E....................................................................................SI...G...............................................................E............................................N............................................................**

**UM0001-4 ......................................D........E...................E................................................................SI...................................................................E.....E....................................................................................M........N.....**

**UM0002-8 ......................................D........E....................................................................................SI...................................................................E.........................................................................................................**

**UM0002-9 ......................................D........E....................................................................................SI...................................................................E.........................................................................................................**

**UM0002-3 ...................................R..D........E....................................................................................SI...................................................................E.............................................................................G...........................**

**UM0002-4 .AT...................................D........E....................................................................................SI......................................A............................E.........................................................................................................**

**UM0002-5 ......................................D........E....................................................................................SI...................................................................E.........................................................................................................**

**MAD-3 ......................................D........E....................................................................................SI........................................V..........................E..................S................................................................................NS....**

**MAD-4 ......................................D..C..........................................................................................SI...................................................................E..................S................................................................................NS....**

**MAD-8 ......................................D........E....................................................................................SIL..G...................S...........................................E..................S................................................................................NS....**

**MAD-8.1 ......................................D........E....................................................................................SI..............................................A....................E..................S................................................................................NS....**

**MAD-9 ......................................D........E....................................................................................SI...................................................................E...K..............S................................................................................NS....**

**MAD-9.1 ......................................D........E....................................................................................SI...................................................................E..................S...................................P.......T....................................NS....**

**MAD-11 ......................................D........E....................................................................................SI..............................................A....................E..................S................................................................................NS....**

**RAU-2 ......................................D........E....................................................................................SI...................................................................E.........................................................................................................**

**RAU-3 ......................................D........E....................................................................................SI...................................................................E........D................................................................................................**

**RAU-7 ......................................D........E....................................................................................SI...................................................................E.........................................................................................................**

**RAU-9 ......................................D........E....................................................................................SI...................................................................E............................................R............................................................**

**UM0002-6 ......................................D........E....................................................................................SI...................................................................E......................T..................................................................................**

**MAH-5 ......................................D........E....................................................................................SI...................................................................E.........................................................................................................**

**MAH-8 ......................................D........E....................................................................................SI...................................................................E............................R............................................................................**

**Monkey569-8 ......................................D........E....................................................................................SI...................................................................E...........................P..............R..............................................................**

**Monkey569-6 ......................................D........E....................................................................................SI...................................................................E...........................P.............................................................................**

**Monkey569-4 ......................................D........E....................................................................................SI...................................................................E...........................P.............................................................................**

**Monkey569-10 ....................N.................D........E................................A...................................................SI...................................................................E...........................P.............................................................................**

**Monkey566-3 ......................................D........E....................................................................................SI...................................................................E.........................................................................................................**

**Monkey566-10 ......................................D........E....................................................................................SI...................................................................E.........................................................................................................**

**Additional file 1: Full amino acid sequence alignment of PkDBPαII.** Amino acid residues identical to those of the reference sequence (strain H), are indicated by dots. The twelve conserved cysteine (C) residues are marked in yellow. The conserved Y94, N95, K96, R103, L168 and I175 residues required for recognition of DARC on human erythrocytes are highlighted in green.
